# Distribution and Composition of Thiotrophic Mats in the Hypoxic Zone of the Black Sea (150–170 m Water Depth, Crimea Margin)

**DOI:** 10.3389/fmicb.2016.01011

**Published:** 2016-06-29

**Authors:** Gerdhard L. Jessen, Anna Lichtschlag, Ulrich Struck, Antje Boetius

**Affiliations:** ^1^HGF-MPG Group for Deep Sea Ecology and Technology, Max Planck Institute for Marine MicrobiologyBremen, Germany; ^2^Museum für Naturkunde, Leibniz-Institut für Evolutions- und BiodiversitätsforschungBerlin, Germany; ^3^Alfred Wegener Institute, Helmholtz Center for Polar and Marine ResearchBremerhaven, Germany

**Keywords:** microbial mats, hypoxia, labile organic matter accumulation, bacterial and archaeal communities, sulfide oxidation, sulfate reduction

## Abstract

At the Black Sea chemocline, oxygen- and sulfide-rich waters meet and form a niche for thiotrophic pelagic bacteria. Here we investigated an area of the Northwestern Black Sea off Crimea close to the shelf break, where the chemocline reaches the seafloor at around 150–170 m water depth, to assess whether thiotrophic bacteria are favored in this zone. Seafloor video transects were carried out with the submersible JAGO covering 20 km^2^ on the region between 110 and 200 m depth. Around the chemocline we observed irregular seafloor depressions, covered with whitish mats of large filamentous bacteria. These comprised 25–55% of the seafloor, forming a belt of 3 km width around the chemocline. Cores from the mats obtained with JAGO showed higher accumulations of organic matter under the mats compared to mat-free sediments. The mat-forming bacteria were related to *Beggiatoa*-like large filamentous sulfur bacteria based on 16S rRNA sequences from the mat, and visual characteristics. The microbial community under the mats was significantly different from the surrounding sediments and enriched with taxa affiliated with polymer degrading, fermenting and sulfate reducing microorganisms. Under the mats, higher organic matter accumulation, as well as higher remineralization and radiotracer-based sulfate reduction rates were measured compared to outside the mat. Mat-covered and mat-free sediments showed similar degradability of the bulk organic matter pool, suggesting that the higher sulfide fluxes and subsequent development of the thiotrophic mats in the patches are consequences of the accumulation of organic matter rather than its qualitative composition. Our observations suggest that the key factors for the distribution of thiotrophic mat-forming communities near to the Crimean shelf break are hypoxic conditions that (i) repress grazers, (ii) enhance the accumulation and degradation of labile organic matter by sulfate-reducers, and (iii) favor thiotrophic filamentous bacteria which are adapted to exploit steep gradients in oxygen and sulfide availability; in addition to a specific seafloor topography which may relate to internal waves at the shelf break.

## Introduction

Thiotrophic microbial mats are dense, visible accumulations of microorganisms, dominated by functional groups capable to gain their energy by using reduced forms of sulfur as electron donors, enabling chemoautotrophic biomass production. They are found at the oxic–anoxic interface of sediments and rocks characterized by high fluxes of sulfide (Macalady et al., 2008; Jørgensen, 2010 and references therein). Typical marine mat-forming thiotrophs inhabiting shelf sediments are “*Candidatus* Maribeggiatoa spp.” (Teske and Nelson, 2006; nomenclature from Salman et al., 2011), “*Ca.* Marithioploca spp.” (Gallardo, 1977; nomenclature from Salman et al., 2011); Thiomargarita spp. (Schulz, 2006; nomenclature from Salman et al., 2011); “*Ca.* Marithrix spp.”; Thiobacterium spp. (Grünke et al., 2011; nomenclature from Salman et al., 2011) and representatives of the family *Thiovulgaceae* (Campbell et al., 2006), as well as some types of *Arcobacter* (Wirsen et al., 2002).

Thiotrophic mats can cover large areas of seafloor in shelf seas and upper continental margins where oxygen depletion leads to high deposition rates of organic matter, such as coastal upwelling regions, oxygen minimum zones and other ecosystems subjected to eutrophication (Jørgensen, 1977; Williams and Reimers, 1983; Schulz and Jørgensen, 2001; Levin, 2003; Mußmann et al., 2003). In these systems, the rapid depletion of oxygen by organic matter remineralization at the seafloor favors high rates of sulfate reduction, and hence sulfide production, which in turn supports the growth of thiotrophs into dense accumulations (Neira and Rackemann, 1996; Freitag et al., 2003; Lehto et al., 2014). Thiotrophic mats are also prominent features of cold seep ecosystems (Grünke et al., 2012), where the anaerobic oxidation of hydrocarbons via sulfate reduction fuels high sulfide fluxes (Boetius et al., 2000; Joye et al., 2004), at sulfide-emitting hydrothermal vents (Jannasch et al., 1989; Meyer et al., 2013; Urich et al., 2014), as well as in caves, profiting from sulfide-rich streams (Macalady et al., 2006).

Among the factors controlling the distribution and thickness of mats in shelf sediments are the fluxes of hydrogen sulfide from the seafloor, and of the electron acceptors oxygen or nitrate from the overlying bottom water (Møller et al., 1985; Schulz et al., 1999; Mußmann et al., 2003; Kamp et al., 2006; Preisler et al., 2007; Grünke et al., 2011). Many of the large sulfur bacteria are able to concentrate and store nitrate in their vacuoles, which they can use as alternative electron acceptor to oxygen, and which helps them to endure fluctuating hypoxic–anoxic conditions (Fossing et al., 1995, Schulz and Jørgensen, 2001; Jørgensen, 2010; Grünke et al., 2011).

The aim of this study was to test if such favorable conditions for the development of thiotrophic mats are met in the Black Sea, especially in its hypoxic zone, where the surface seafloor is in contact with the Black Sea chemocline. Here, the permanent stratification of the water column by a gradient in salinity separates the fresher, oxic surface layer from the permanently anoxic and sulfidic deep water mass (Ross et al., 1970; Murray et al., 2007). At the interface, a chemocline forms, were oxygen and sulfide may co-exist in dynamic equilibrium (Sorokin, 1972; Karl, 1978; Jørgensen et al., 1991). In the water column, the chemocline favors the growth of thiotrophic pelagic bacteria, such as *Gammaproteobacteria* of the SUP05 cluster (Glaubitz et al., 2013). The Black Sea chemocline persists at about 100 m in open waters, and deepens towards the margin to depths of 150–160 m (Stanev et al., 2014). Where it meets the seafloor, it exposes the organic rich sediments to variable hypoxic to anoxic conditions (Friedrich et al., 2014). Just below the chemocline, sulfate reduction becomes the dominant remineralization pathway (Jørgensen et al., 2001) increasing from 50 up to 100% of the total mineralization of organic matter (Weber et al., 2001). This suggests that thiotrophic bacteria could be favored at the interface between the chemocline and the seafloor of the Black Sea outer shelf, as their water column counterpart (Vetriani et al., 2003; Glaubitz et al., 2013). However, previous to this study, the presence and distribution of thiotrophic mats had not been investigated in the Black Sea. Moreover, considering that the development of thiotrophic is one of the most evident response of the microbial community to eutrophication and hypoxia, a better understanding of its distribution and function may enable better predictions of how microbial communities may change with the projected spread of hypoxia and dead zones in the Black Sea and worldwide.

Here we studied the outer Western Crimean shelf by combining submersible surveys with high-resolution geochemical and microbiological analyses, to test the hypothesis that thiotrophic mats could form at the chemocline in hypoxic areas of the Black Sea margin. Key questions addressed were: (i) what are the dominant factors that govern the development of microbial mats? (ii) which microbial types may form mats in the Black Sea, and (iii) what are be the community structure and activity of the mats?

## Materials and Methods

### Study Area and Seafloor Sampling

The Black Sea is a semi-enclosed inland sea, and it is the largest natural anoxic water body in the world. The continental shelf reaches about 40 km from the coast to the shelf break at 100–150 m depth and the Northwestern Shelf (western Crimea) covers more than 90% of the total area of the Black Sea shelf (Panin and Jipa, 2002). This zone also receives most of the massive fluvial discharge, which appears as the main driver of eutrophication and hypoxia on the Northwestern Shelf (Capet et al., 2013).

**FIGURE 1:**
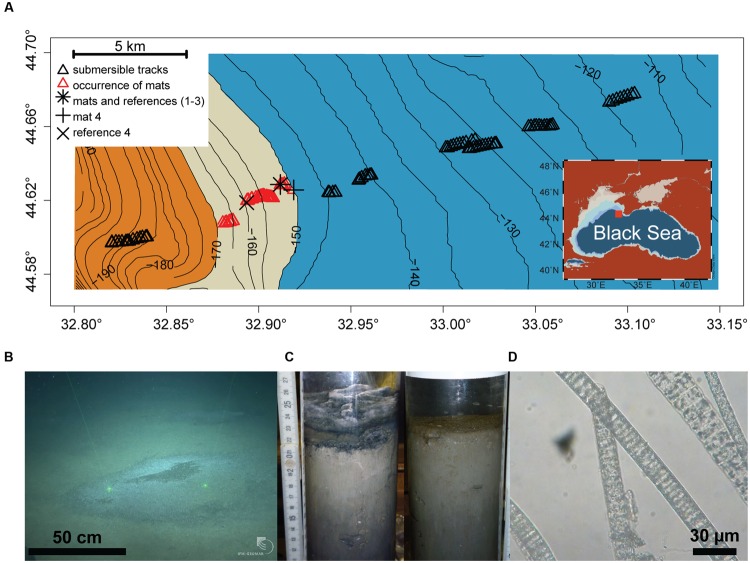
**(A)** Study area depicting the chemocline (beige belt between 150 and 170 m depth) between the oxic (blue) and anoxic/euxinic conditions (orange) on the Crimea outer shelf. Submersible dive tracks are represented by black triangles, and the occurrence of microbial mats by the red triangles (see **Supplementary Figure S2** for further for details about mat size and distribution). **(B)** JAGO-based image of accumulated organic matter covered by microbial mats (copyright IFM-GEOMAR), the distance between the two laser pointers is 50 cm. **(C)** Push cores from mat-covered sediments (left) and reference site (right). **(D)** Light microscopy image of the mat-forming filamentous bacteria, showing sulfur inclusions.

The study area (44.57–44.70° N and 32.80–33.15°E, 110–200 m depth) was located on the Crimean shelf break of the Northwestern Black Sea. Sampling was performed during the MSM 15-1 expedition (R/V Maria S. Merian, 12th April–8th May 2010; **Figure 1A**). In order to assess presence and distribution of microbial mats, 10 video transects were carried out with the submersible JAGO (20 h of video footage, ∼2 h per dive), covering the region between 110 and 200 m depth, and an area of 20 km^2^ (**Figure 1A**). Mats were identified visually by their whitish color, and their dimensions were measured by the laser pointers of JAGO. We observed that the mats were associated with dark patches of particles accumulating in seafloor depressions associated with the shelf break. Mats and underlying sediments, as well as mat free sediments were retrieved either by push coring (PUC, inner diameter of 72 mm) with the manipulator of the submersible JAGO or with a video-controlled multiple corer (TV-MUC, inner diameter of 96 mm). Cores were collected from four different microbial mats, and from three reference sites a few meters away from the mats, on mat-free seafloor outside of depressions (**Table 1**). An additional sediment core was taken as reference for porewater geochemistry (station ref 4). All sediments were sampled on 4th May 2010 with PUCs except “mat 4” and “ref 4”, sampled with a TV-MUC on 26th and 27th April 2010, respectively.

**Table 1:** Biogeochemical composition of mat-covered sediments (mat) and mat-free reference sites.

Sampling site ID	Location (Lat/Long)	Depth (m)	C_org_(%dw)	TN (%dw)	δ^13^C	C_org_:N	Chl *a*: CPE	DI
mat 1	44.62696667° N	152	4.4 ± 0.5	0.6 ± 0.2	-24.6	8.3 ± 0.9	0.4	1.2
	32.91266333° E							
mat 2	44.62710167° N	152	2.9 ± 0.8	0.3 ± 0.2	-24.4	9.4 ± 1.3	0.4	1.5
	32.912615° E							
mat 3	44.62740167° N	152	4.1 ± 0.0	0.5 ± 0.0	-24.6	8.7 ± 0.2	0.4	–
	32.91257167° E							
mat 4	44.62583333° N	156	5.9 ± 0.9	0.6 ± 0.3	-24.2	9.6 ± 0.2	0.5	1.2
	32.91633333° E							
ref 1	44.62696667° N	152	5.0 ± 0.1	0.6 ± 0.0	-24.2	9.9 ± 0.4	0.4	1.6
	32.91266667° E							
ref 2	44.62711667° N	152	1.7 ± 0.4	0.2 ± 0.0	–	8.0 ± 0.4	0.3	1.1
	32.912615° E							
ref 3	44.62718167° N	152	3.8 ± 0.2	0.3 ± 0.0	-24.7	8.8 ± 0.6	0.5	1.3
	32.912615° E							


*Organic carbon (C_*org*_ % dw), nitrogen (TN % dw), carbon stable isotopic composition (*δ^13^C_org_ ‰*), molar organic carbon to nitrogen ratio (C_*org*_:N), chlorophyll a to chloroplastic pigment equivalents ratio (Chl *a*: CPE) and quantitative amino-acid degradation index (DI). Values represent average and standard deviation (±, *n* = 3–5) when available, –: no data.*

Sampling points and respective labels are summarized in **Table 1** and **Figure 1A** comprehensive characterization of the pore water geochemistry and sediment properties beyond the summary in **Table 1** can be found at the Earth System database www.PANGAEA.de.^1^

### Sensor Measurements

To assess oxygen concentrations, temperature and salinity in the bottom waters, a CTD-cast profile (SBE 911plus system, with additional sensors for oxygen-SBE43) was taken before TV-MUC sampling. In case of the JAGO dives, bottom water oxygen concentration was continuously recorded by an oxygen optode (4330 Mk2 AANDERAA optode, detection limit 1 μmol L^-1^) fixed to the frame of the submersible. The optode was calibrated with water samples in which the oxygen concentration was determined by Winkler titration (Winkler, 1888).

High-resolution geochemical gradients of H_2_S and pH were measured *ex situ* with microsensors (Jeroschewski et al., 1996; de Beer et al., 1997) in undisturbed mat-covered sediments (station mat 1), and in sediments without mat within 24 h after collection. Sediments were kept at *in situ* temperature and were capped until the measurements (to avoid oxygen intrusion). The H_2_S sensors were calibrated by stepwise increasing H_2_S concentrations by adding aliquots of a 0.1 mol L^-1^ Na_2_S solution to acidified seawater (pH < 2). pH sensors were calibrated with commercial laboratory buffers. The total sulfide (H_2_S +HS^-^ + S^2-^) was calculated from the H_2_S concentrations and the local pH using equilibrium constants. The sensors were mounted on a motor-driven micromanipulator and lowered into the water column and the sediment in increments of 100 μm. The data acquisition was performed using a DAQ-PAD 6015 (National Instruments Corporation, Austin, TX, USA) and a computer and the flux calculations were performed according to Lichtschlag et al. (2010).

### Pore Water Geochemistry

Pore water from a mat-covered sediment and a reference site was extracted with Rhizons in one cm intervals (Rhizosphere Research Products, pore size <0.2 μm) according to Seeberg-Elverfeldt et al. (2005) (station mat 4 and ref 4; **Figure 1A**). The initial 0.5 mL was discarded and less than 4 mL were extracted per Rhizon to prevent mixing between horizons. The retrieved pore water was immediately sub-sampled and fixed in 20% Zn acetate (total H_2_S), or frozen at -20°C (${NH}_{4}^{+}$ and ${PO}_{4}^{3-}$) for further analysis. ${NH}_{4}^{+}$, ${PO}_{4}^{3-}$ and total H_2_S were measured spectrophotometrically using the method of Grasshoff et al. (1983) for nutrients and Cline (1969) to assess total H_2_S (methylene blue).

In absence of oxygen, sulfate reduction is the most dominant carbon mineralization pathway on the hypoxic–anoxic Crimean shelf break area (Lichtschlag et al., 2015). Thus carbon mineralization was assessed via sulfate reduction and calculated based on stoichiometry according to Thamdrup and Canfield (1996) as follows:

$$2{CH}_{2}O+{SO}_{4}^{2-}\to {2HCO}_{3}^{-}+H_{2}S$$

### Bulk-Sediment Analyses

Immediately after retrieval, sediments were sliced into 1-cm-thick subsamples in a cold room set to *in situ* temperature (8°C), and processed for further analyses.

To asses organic matter composition and degradability, samples for organic carbon (C_org_) and total nitrogen, pigments (chlorophyll *a* and its degradation products; summed up as chloroplastic pigment equivalents, CPE) and total hydrolyzable amino acids (THAA) were measured in triplicate from freeze dried and homogenized sediment.

Pigments (chlorophyll *a* and CPE) were measured spectrophotometrically according to Schubert et al. (2005). THAA were measured after Pantoja and Lee (2003) from ca. 100 mg sediment. Briefly, after hydrolysis (6 N HCl at 105°C, for 21 h under N_2_ gas), the supernatant was removed and neutralized (0.1 N KOH). The amino acid identification and quantification was conducted by high performance liquid chromatography (HPLC) after pre-column derivatization with o-phthaldialdehyde and 2-mercaptoethanol (Lindroth and Mopper, 1979; Pantoja and Lee, 1999). The results obtained were used to assess the degradation index (DI) of the organic matter, which represents the selective diagenetic alteration of the sedimentary amino acids as a proxy of lability of the organic matter (Dauwe and Middelburg, 1998; Dauwe et al., 1999). Porosity was determined as the loss of water content of a known volume of sediment before and after drying at 60°C until constant weight.

Stable isotope analysis of organic carbon and concentration measurements of nitrogen and carbon were performed simultaneously with a THERMO Delta V isotope ratio mass spectrometer, coupled to a THERMO Flash EA 1112 elemental analyzer via a THERMO Conflo IV-interface. Before analysis, inorganic carbon was removed by acidification (12.5% HCl solution). Stable isotope ratios are expressed in per mill (‰), the conventional delta notation (δ^13^C) relative to VPDB (Vienna PeeDee Belemnite standard). Standard deviation for repeated stable isotope measurements of lab standard material (peptone) is generally better than 0.15 (‰) for carbon. Standard deviations of concentration measurements of replicates of our lab standard are <3% of the concentration analyzed.

### Sulfate Reduction Rates

Rates of sulfate reduction (SR) were measured by the whole core injection method (Jørgensen, 1978; Kallmeyer et al., 2004) at stations mat 4 and ref 4. Here sediments were vertically sub-sampled with subcore liners (250 mm inner diameter) in triplicate and injected with ${{}^{35}SO}_{4}^{2-}$ radiotracer. After 12 h incubation in the dark at *in situ* temperature, the reactions were stopped by transferring the core slices (1 cm) into 20% Zn acetate.

### Microbial Community Characterization

Bacterial filaments and the accompanying communities were assessed by direct light microscopy and the length and width of the filaments were measured using a calibrated ocular micrometer. The total number of cells in the underlying sediments, and in sediments next to mats were assessed in the home laboratory by the Acridine Orange Direct Count (AODC) method. Subsampled sediments were fixed in 2% formaldehyde/filtered artificial seawater, stored at 4°C and treated according to Velji and Albright (1986) and Boetius and Lochte (1996). Single cell numbers were determined by randomly counting at least 30 grids per filter (for two replicate 0.02 μm pore size filters).

Total DNA was extracted from ca. 1 g samples of wet sediment from core sections under the thiotrophic mat, or from sediments next to it, and stored at -20°C using UltraClean Soil DNA Isolation Kits (MoBio Laboratories Inc., Carlsbad, CA, USA). Extracted DNA was quantified with a microplate spectrometer (Infinite^®^ 200 PRO NanoQuant, TECAN Ltd, Switzerland) and its concentration adjusted for each step of the subsequent molecular protocol. The bacterial community structure was determined by the automated ribosomal intergenic spacer analysis (ARISA) fingerprinting method according to Fisher and Triplett (1999). Triplicate PCR reactions from standardized amounts of DNA (10 ng) from each sample were amplified using the bacteria forward FAM-labeled primer (ITSF: 5′-GTCGTAACAAGGTAGCCGTA-3′) and reverse primer (ITSReub: 5′-CCAAGGCATCCACC-3′) (Cardinale et al., 2004). Fluorescently labeled fragments were determined on a capillary sequencer, and only ARISA fragments in the size range of 100–1000 base pairs (bp) were binned into operational taxonomic units (OTUs) using custom R scripts^2^ (v. 3.0.1) available at http://www.mpi-bremen.de/en/Software_4.html.

Extracted DNA was amplified and sequenced by the Research and Testing Laboratory (Lubbock Texas, USA) via 454 Massively Parallel Tag Sequencing (454-MPTS). The V4–V6 region of the 16 S rRNA genes were amplified using the bacterial primers 530F (5′-GTGCCAGCMGCCGCGG-3′) and 1100R (5′-GGGTTGCGCTCGTTG-3′), and the archaeal primers 349F (5′-GYGCASCAGKCGMGAAW-3′) and 1048R (5′-CGRCRGCCATGYACCWC-3′) according to Klindworth et al. (2013). Fragments were sequenced following the 454-MPTS protocol (Margulies et al., 2005) and Titanium reagent chemistry. The raw sequences were processed with *mothur* (v. 1.29) following a rigorous quality control procedure including denoising of the flowgrams using an algorithm based on *PyroNoise* (Quince et al., 2009) removal of PCR errors and a chimera check using *UCHIME* (Edgar et al., 2011) and average neighbor clustering algorithm (Schloss et al., 2009, 2011, see Supplementary Material for workflow details). Taxonomic assignments were carried out using the SILVA reference file (Pruesse et al., 2007; downloaded from http://www.mothur.org in September 2013) and clustered at a 97% identity level into operational taxonomic units (OTU_0.03_), and microbial function was inferred from cultured representatives of the identified sequences. The dataset was normalized by the total amount of sequences per sample to get relative abundances, and singletons were removed. 16S rRNA gene sequences of filamentous bacteria are known to be underrepresented in full sediment DNA extracts during PCR, due to epibiont contamination (e.g., Schulz, 2006), and the abundance of sediment-associated microbes (Girnth et al., 2011). Sequences obtained in this study were deposited at EMBL under the project accession number PRJEB11179.

### Statistical Analysis

All statistical analyses were conducted following Ramette (2007 and references therein) using the R package vegan (Oksanen et al., 2010) and custom R scripts available at http://www.mpi-bremen.de/en/Software_4.html. The difference in organic matter availability between mat and reference site was assessed by a Student’s *t*-test, while the Jaccard dissimilarity index was used to calculate the number of shared OTUs between samples. The bacterial community structure was visualized by applying Nonmetric Multi Dimensional Scaling (NMDS) analysis from Bray–Curtis dissimilarity of relative OTU abundances. Separations of groups identified on the NMDS plot (mat and reference) were tested for significance using the non-parametric Analysis of Similarity (ANOSIM) test and *P*-values were corrected for multiple comparisons using Bonferroni’s correction.

## Results and Discussion

Dives with the submersible JAGO confirmed our hypothesis that thiotrophic microbial mats are associated with the hypoxic zone of the Black Sea at the outer shelf break. We defined the hypoxic zone according to a widely accepted ecological threshold (e.g., Diaz, 2001), as the area marked by bottom water oxygen concentrations 1–63 μmol L^-1^ O_2_. In the outer Western Crimean shelf this zone ranged from ∼140 to 170 m water depth (with a rather uniform bottom water salinity of 20 psu, and a temperature of 8°C), covering a distance of ca. 6 km perpendicular to the slope, as detected by the oxygen optode mounted to JAGO (**Figure 1A**). No larger benthic fauna was observed to graze the seafloor within this zone during 10 dives, covering about 20 km^2^, and the seafloor did not show signs of animal traces such as burrows.

Within this area, mats were directly associated with the chemocline, i.e., the interface between oxic and sulfidic bottom waters. This zone was positioned on average at 160 m water depth during our investigations in late April–early May 2010. However, *in situ* observations indicate that this zone is dynamic and fluctuates up and down, for up to 1 km due to internal waves and eddies (Filonov, 2000; Friedrich et al., 2014; Stanev et al., 2014; Lichtschlag et al., 2015). During the dive, the observed oxygen concentrations in bottom waters of the mat area ranged from below detection limit (<1 μmol L^-1^) at mats 1–3 to ca. 50 μmol L^-1^ at mat 4, as measured by JAGO and CTD casts. Studies of the faunal composition of the area showed a steep decrease in faunal abundances and absence of dwelling animals within this zone (Lichtschlag et al., 2015). Only very small polychaetes (<3 mm genus *Victorniella*) and some foraminifera (e.g., genus *Vellaria*) were detected around 130–170 m (Sergeeva, 2015; Sergeeva et al., 2015). Highly adapted sub-millimeter sized metazoans have been reported active previously in anoxic waters of the Bosphorus outflow area (e.g., *Theristus* sp.; Sergeeva et al., 2014) and other euxinic environments (e.g., Phylum *Loricifera*; Danovaro et al., 2010). However, these conditions are generally inhospitable to larger metazoans which dwell the seafloor and provide critical ecosystem functions such as bioturbation and sediment feeding (e.g., Levin, 2003, 2010 and references therein).

### Thiotrophic Mats Associate with Organic Matter Accumulations at the Chemocline

**FIGURE 2:**
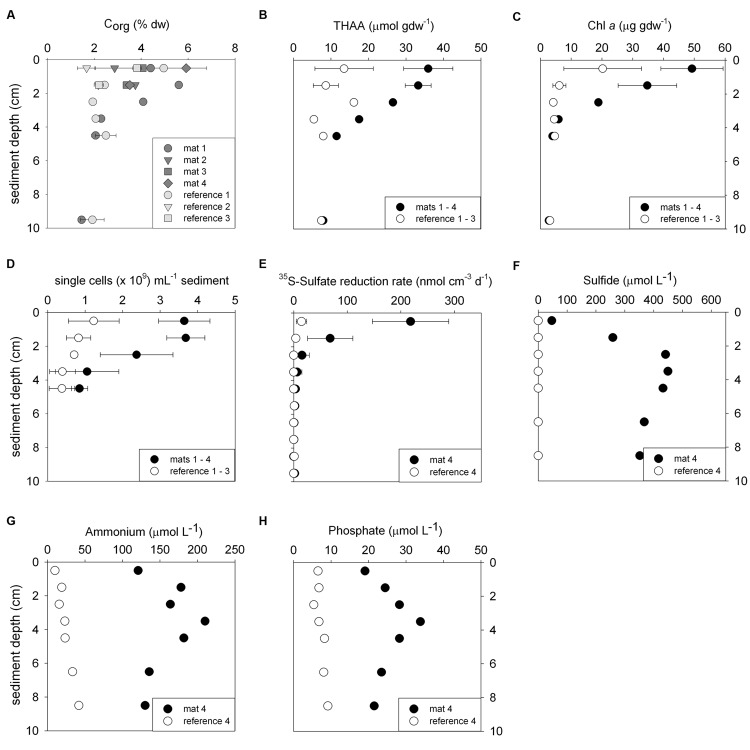
**Biogeochemical characteristics of the sediments comparing mat-covered (dark gray/black symbols) and mat-free references sites (light gray/white symbols).** Content of organic carbon **(A)**, total hydrolyzable amino acids (THAA) **(B)** and chlorophyll *a* **(C)**, total single cells **(D)**, radiotracer-based sulfate reduction rates **(E)**, concentration of sulfide **(F)**, ammonium **(G)** and phosphate **(H)**. Symbols represent averages and error bars standard deviations when replicates were available (±, *n* = 3–5).

The submersible surveys close to the position of the chemocline showed a color change of the seafloor from beige-brownish to dark-gray with increasing water depth. The seafloor was covered with a fluffy layer of greenish detritus particles, which were substantially enriched in algal pigments (**Figure 2C**), suggesting that this layer was composed of deposited marine snow. Cutting into the mat-covered patches with the JAGO manipulator or with push cores showed laminated, almost gelatinous layers of particle deposits of around 4–5 cm thickness that filled the seafloor depressions (**Figure 1C**). In contrast, the particle layer outside the mats was only around 1 cm thickness above a flat seafloor. The seafloor depressions ranged from few decimeters to >3 m in diameter, and the darker patches of organic matter accumulations formed a slightly elevated dome within them. Using the JAGO arm for measurements *in situ*, and comparing to the thickness of the particle layer in the retrieved core, we estimate the depth of the depressions at around 4 cm. They were covered partially or fully by mat-forming, whitish microbial filaments (**Figure 1B**). Most of the mats were <1 m in diameter (0.6 ± 0.3 m; *n* = 1091), and rather homogeneously distributed with a few meters apart from each other (**Supplementary Figure S2**). The origin and distribution of the abundant depressions observed close to the chemocline at the shelf break is unknown. It may be possible that these are formed by the impact of currents by internal waves and eddies in the region (Trembanis et al., 2011; Stanev et al., 2014).

No mats were observed below the chemocline within oxygen-free sulfidic waters at >170 m water depth, or above the 150 m isobath where the seafloor depressions were rare (**Figure 1**; **Supplementary Figure S2**). From the dimension and distribution of the mats, we estimate that they covered an area in the range of 25–55% of the explored seafloor between 150 and 170 m water depth. Extrapolating from our observations, the Black Sea outer shelf may host a belt of mat patches between these two isobaths, covering an area of 500–1200 km^2^ of Black Sea seafloor (**Supplementary Figure S1**).

### Accumulation of Organic Matter Prompts Sulfide Production for Mat Development

**FIGURE 3:**
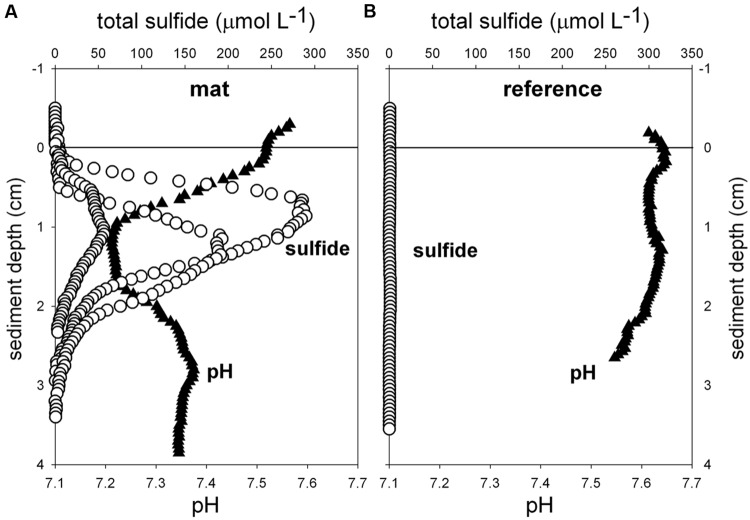
***Ex situ* microprofiles of total sulfide (*n* = 3) and pH (*n* = 1) measured in sediment of mat 1 **(A)**, and in close-by sediments without mat (**B**, *n* = 1).** Oxygen was below detection limit.

Biogeochemical analyses of sediment samples from the mat-covered vs. mat-free seafloor confirmed the enhanced accumulation of organic matter underlying the mats compared to the surrounding sediments (**Figures 2A–C**). The pore waters from sediments with mats contained sulfide concentrations up to 450 μmol L^-1^, and microsensor measurements showed fluxes of 0.7–3.2 mmol sulfide m^-2^ day^-1^ (average 1.9 mmol sulfide m^-2^ day^-1^), consistent with sulfate reduction rate measurements (average 2.2 mmol sulfide m^-2^ day^-1^) and porewater analyses (**Figures 2E,F**). Outside the mats, sulfide concentrations and fluxes were around or below detection limit, whether measured as sulfate reduction rates, with microsensors (upper 4 cm, **Figure 3**) or in the extracted porewater (down to 10 cm, **Figure 2F**). The bottom water sulfide concentrations at these depths were around detection limit (Lichtschlag et al., 2015), depending on the position of the chemocline and the hydrographical regime. Our observations indicate that the occurrence of thiotrophic mats is limited to a zone in the Black Sea with both oxygen availability and porewater sulfide fluxes high enough to reach into the surface seafloor (**Figure 3**). Indeed, the steep gradient of the sulfide microprofiles within the mat is consistent with the complete removal of sulfide (Lichtschlag et al., 2010).

The observed organic matter accumulations and porewater profiles in the depressions were also reflected in enhanced remineralization rates. Assuming carbon degradation was entirely fueled by sulfate reduction, total microbial remineralization below the mat was 5–10 mmol C m^-2^ day^-1^ based on the stoichiometry with sulfide production compared to rates of 1.3 ± 0.5 mmol C m^-2^ day^-1^ in the mat-free hypoxic area (Lichtschlag et al., 2015). In the anoxic zone of the shelf below the chemocline, sulfate reduction rates remain high (Weber et al., 2001; Lichtschlag et al., 2015), but the lack of oxygen represses the formation of mats.

Next we investigated the reason for the enhanced carbon and sulfur fluxes in the seafloor depressions. The organic carbon (C_org_) content of the surface seafloor (top 1 cm) from the mat patches and outside of the mats were highly variable and fell within the range described for the Black Sea of 1–5% (e.g. Cowie and Hedges, 1991; Jørgensen et al., 2004). When integrating over the top 2 cm; the patches showed significantly higher C_org_ compared to the surrounding reference sites averaging 4.5 ± 1.5% vs. 3.5 ± 1.5% outside the mat [*t*_(32)_ = 3.48, *P* < 0.001]. Within the top 4 cm this even resulted in ca. twofold higher C_org_ values (**Figure 2A**). Also the percentage of total nitrogen integrated over the same section was elevated. However, the isotopic composition of C and N was the same inside and outside the mat (**Table 1**), suggesting a similar source of the deposited organic matter. Furthermore, both chloroplastic pigments from algal detritus and total hydrolyzable amino acids showed higher concentrations under the mat compared to the reference sites (**Figures 2B,C**), indicating phytodetritus accumulation in the seafloor depressions. We also tested for differences in the degradability of the bulk organic matter pool accumulating in the depressions versus on the surrounding seafloor by a number of proxies based on the above concentration measurements. This included the C/N molar ratio (Meyers, 1994), the amino acid based degradation index (Dauwe and Middelburg, 1998; Dauwe et al., 1999) and the chlorophyll *a* to total pigment equivalent (CPE) ratio (Pfannkuche, 1993). The respective proxies were comparable between mat-covered sediments and the reference sites (**Table 1**), suggesting that local differences in the accumulation rates of matter, but not the quality and composition of the material, was responsible for the higher sulfide fluxes and subsequent development of microbial mats. Considering sediment accumulation rates of 1 ± 0.5 mm year^-1^ for the study area (Lichtschlag et al., 2015), apparently, the depressions in the seafloor act as detritus traps. From the shape of the sulfide, ammonium and phosphate profiles these appeared as rather recent deposits not yet in steady state (**Figures 2F–H**).

### Composition and Diversity of the Thiotrophic Mats on the Crimea Margin

**FIGURE 4:**
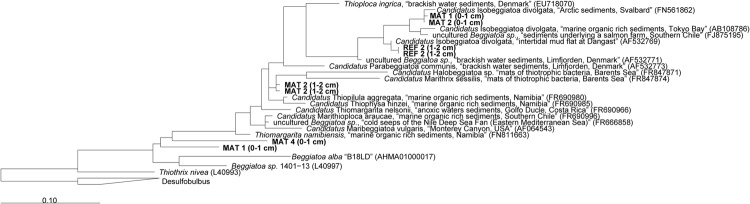
**Phylogenetic 16S rRNA gene based tree showing the affiliation of sequences extracted from mat-covered and reference sediments of the Crimea margin with other filamentous sulfur-oxidizing bacteria.** Mats and references sequences were screened for relatives of large sulfur oxidizing bacteria and extracted from the data set using the “grep” function in R. Initial tree reconstruction was conducted with nearly full-length sequences (1200–1400 bp). Partial sequences were subsequently inserted into the reconstructed consensus tree by applying the parsimony criteria. The scale bar corresponds to 10% estimated sequence divergence. Phylogenetic classification was carried out with the software package ARB (Ludwig et al., 2004) based on the SILVA database (SSURef v119, release date: 24 July 2014). The phylogenetic tree was calculated with the maximum likelihood algorithm PHYML (100 bootstraps) as implemented in ARB using a positional variability filter.

The mats forming above the detritus-accumulating depressions consisted of colorless filaments ranging from 10 to 40 μm diameter and up to a few mm length. The filaments contained light refracting granules of elemental sulfur (**Figure 1D**), explaining the whitish appearance of the mats. According to the morphology and the occasional gliding motility, many filaments resembled a *Beggiatoa*-like morphotype (Schulz and Jørgensen, 2001; nomenclature from Salman et al., 2011). Other filaments were seemingly non-motile under the microscope and rather represented the morphotype of “*Candidatus* Marithrix” (Kalanetra et al., 2004; nomenclature from Salman et al., 2011). We retrieved 16S rRNA gene sequences from the top sediment layer (0–2 cm) affiliated to the group of large filamentous sulfide oxidizers (**Figure 4**). The sequences from the mat clustered with relatives of the free-living, gliding “*Candidatus* Isobeggiatoa divolgata” (Mußmann et al., 2007), with the attached-living “*Ca.* Marithrix sp.”, and other deeply branching thiotrophs within the family *Beggiatoaceae*. Surprisingly, in the mat-free reference site 2 (**Figure 4**) we also observed “*Ca*. I. divolgata” sequences, which indicates that some of the thiotrophs can be wide-spread in the Black Sea sediments as a member of the rare biosphere, and could grow into dense mats when they meet favorable conditions (Jørgensen, 2010).

We used cell counts and community fingerprinting (ARISA and 454-MPTS) to assess the distribution and composition of the bacterial and archaeal communities associated with the thiotrophic mats. Since the total DNA pool was sequenced, a fraction of extracellular DNA and dormant/dead cells can be represented in addition to the active microbial community (Torti et al., 2015 and references therein).

**FIGURE 5:**
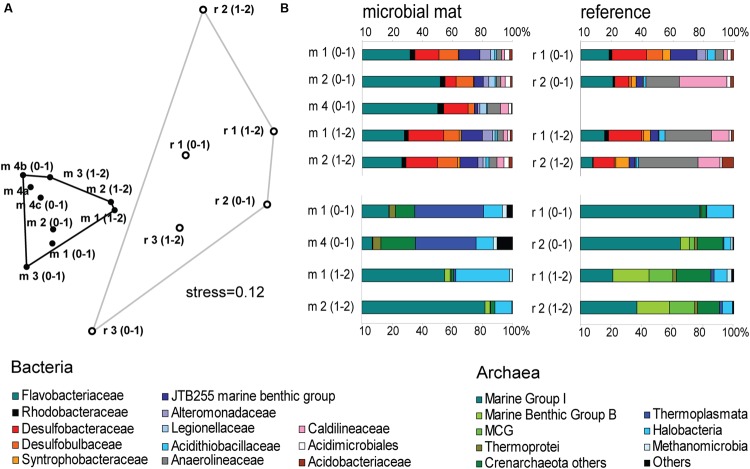
**(A)** Nonmetric Multi Dimensional Scaling (NMDS) ordination plot based on Bray–Curtis distance matrix from ARISA profiles. Convex hulls depict significant differences between groups of mat-covered sediments (black circles) and references sites (white circles). Bonferroni corrected *P*-value < 0.001; Analysis of Similarity (ANOSIM) based on Bray–Curtis *R*-value = 0.7. **(B)** Relative abundance of bacterial (above) and archaeal (below) sequences from the upper 2 cm (0–1 and 1–2 cm horizons) obtained by 454-MPTS comparing mat-covered sediments (left) and references sites (right). MCG-Miscellaneous Crenarchaeotal Group.

The mat-associated communities showed threefold higher cell abundances in the upper sediment horizons (0–4 cm b.s.f.) compared to the surrounding sediments (**Figure 2D**). Significant differences in the bacterial community structure were evident based on the ARISA and the MPTS data comparing mat and reference sites (ARISA Bonferroni corrected *P*-value < 0.001; Analysis of Similarity based on Bray–Curtis *R*-value = 0.7; **Figure 5A**), consistent with the variations in the composition of bacterial and archaeal taxa detected by 454-MPTS (upper 2 cm; MPTS Bonferroni corrected *P*-value < 0.05; Analysis of Similarity based on Bray–Curtis *R*-value = 0.6; **Figure 5B**). Within the same horizon, only 45 ± 6% of the dominant bacterial types detected by ARISA overlapped between bacterial mats and surrounding reference sediments (**Figure 5A**; **Supplementary Table S1**), and the thiotrophic mats hosted 11% unique sequences.

The Black Sea microbiological communities associated with hydrocarbon-rich benthic ecosystems are relatively well studied. However, apart from these environments the diversity of Black Sea benthic microbial communities remains less understood. From the 454-MPTS analysis of the composition of bacterial sequences in mat-covered and surrounding sediments were retrieved 64429 reads, comprising 3766 individual OTUs by 97% clustering (excluding singletons), in the following referred to as OTU_0.03_ (**Supplementary Table S2**).

*Flavobacteriaceae* dominated up to 50% of the individual OTU_0.03_ at the surface of the patches (**Figure 5B**). *Flavobacteria* are relevant marine polymer degraders with hydrolytic and fermentative capacities (Cottrell and Kirchman, 2000; Bernardet et al., 2002 and references therein; Lydell et al., 2004; Julies et al., 2010). Their sequence abundance was two-to fourfold higher in mat-covered sediments compared to the surrounding sediments, and generally decreased with sediment depth and with decreasing organic matter content, as observed already in the Black Sea (Romanian shelf; Tanase et al., 2009) and other marine habitats (Bissett et al., 2008). Probably profiting from the products of fermentation, the families of sulfate/sulfur reducers *Desulfobacteraceae* and *Desulfobulbaceae* (Muyzer and Stams, 2008) were more abundant in the mat-covered sediments compared to the surrounding sediments. These groups are typical for reduced sediments (Reese et al., 2013; Sinkko et al., 2013). Previously found associated to *Beggiatoa* spp. mats (Lloyd et al., 2010), appeared as the most abundant among sulfate reducing bacteria in Black Sea surface sediments at similar water depth (Ince et al., 2007). Some members of the *Desulfobulbaceae* are able to conduct long range electron transfer in organic rich sediments, coupling the oxidation of sulfide with oxygen over centimeter distance (Pfeffer et al., 2012). Several of the 16SrRNA gene sequences retrieved from the mat clustered with sequences identified as “cable bacteria” (**Supplementary Figure S3**, Pfeffer et al., 2012; Schauer et al., 2014), extending the known distribution of this group (Malkin et al., 2014). However, the lack of the characteristic pH signature (**Figure 3**) suggest they were rare, and that sulfate reduction was a main energy-generating pathway, probably from phytodetrital fermentation products (McKew et al., 2013). Two fractions of the bacterial community did not show differences between mat and mat-free sediments, the families *Anaerolineaceae* and *Caldilineaceae* involved in fermentation (Yamada et al., 2006) and previously described for deep Black Sea sediments (>800 m; Schippers et al., 2012).

Also the archaeal community (39091 reads retrieved, comprising 880 individual OTUs (excluding singletons); 97% clustering, **Supplementary Table S2**) showed differences between mat-covered sediments and the surrounding seafloor (**Figure 5B**). *Thermoplasmata* were typical for the mat-covered sediments, but almost absent from the surrounding sediments. They are known as facultative anaerobes performing sulfur respiration (Huber and Stetter, 2006 and references therein), suggesting a role in the consumption of sulfide (**Figure 3**). *Crenarchaeota* sequence abundances were low in the sulfidic mat-covered sediments, but were the most abundant archaeal sequence elsewhere, extending its dominance from deep Black Sea sediments (>800 m; Schippers et al., 2012) to the continental shelf. Many of the archaeal OTU_0.03_ were affiliated to the Marine Group I. This clade dominated subsurface sediments at the mat-covered sediments and also the surface and subsurface of the references sites (**Figure 5B**). Cultivated strains affiliated to this group are described as aerobic ammonia oxidizers (Könneke et al., 2005). In agreement with our results they can inhabit oxic and anoxic environments, but little is known as to which electron acceptors they could use in the absence of oxygen (La Cono et al., 2013). In addition, we detected several types associated with the thiotrophic mats belonging to “extremophile” groups (**Figure 5B**), including *Thermoprotei* and *Halobacteria* considered thermophilic and halophilic correspondingly (Huber et al., 2006; Oren, 2006 and references therein). Although most of the representatives of the *Halobacteria* group are halophiles, uncultured members have been reported in less saline environments such as estuaries (e.g., Singh et al., 2010). *Halobacteria* were more abundant in the mat-covered sediments; they are known to reduce sulfur compounds in low-salt environments (Elshahed et al., 2004), however, the bottom waters presented *S* = 20 during the study.

## Conclusion

We could confirm our hypothesis of an association of thiotrophic mats with the hypoxic areas of the Black Sea margin, specifically the zone where the chemocline met the seafloor. The thiotrophic mats were formed by filamentous large sulfur bacteria related to the *Beggiatoa* clade. The mat habitat enriched for polymer degrading, fermenting and sulfate-reducing microorganisms including relatives of the cable bacteria. The key factor for the distribution of mat-forming thiotrophs appeared to be the proximity to the chemocline, for access to oxygen from bottom waters, and sulfide from the sediment, and for reduced grazing pressure due to the absence of fauna in hypoxic waters. We speculate that also the specific hydrographic conditions near to the shelf break are important in shaping the habitat, causing a rough seafloor topography with depressions that enhance the accumulation of relatively labile organic matter. Due to the dynamic positioning of this interface zone by water mass movement, we suggest that the thiotrophic mat zone could form a belt around the Black Sea outer shelf, covering thousand square kilometers.

## Author Contributions

Conception, work design, and drafting by GLJ and AB. Acquisition, analysis and data interpretation by all the authors. All the authors revised the work critically and contributed with important intellectual content, approved it for publishing and agreed for all aspects of the work in ensuring that questions related to the accuracy and integrity of any part of the work were appropriately investigated and resolved.

## Conflict of Interest Statement

The authors declare that the research was conducted in the absence of any commercial or financial relationships that could be construed as a potential conflict of interest.

## Footnotes

1 http://doi.pangaea.de/10.1594/PANGAEA.858227

2 http://www.R-project.org
