# Supplementary material for: Distribution and Composition of Thiotrophic Mats in the Hypoxic Zone of the Black Sea (150–170 m Water Depth, Crimea Margin)
Source: Front Microbiol. 2016 Jun 29;7:1011. doi: 10.3389/fmicb.2016.01011 (PMC4925705; doi:10.3389/fmicb.2016.01011)
Supplement: Supplementary file 1 [file Table_1.PDF]

Supplementary Table 1. Percentage of shared OTU (from ARISA profiles) between mat-covered sediments and reference sites

	**mat 1 (0-1)**	**mat 1 (1-2)**	**mat 2 (0-1)**	**mat 2 (1-2)**	**mat 3 (0-1)**	**mat 3 (1-2)**	**mat 4a (0-1)**	**mat 4b (0-1)**	**mat 4c (0-1)**	**ref 1 (0-1)**	**ref 1 (1-2)**	**ref 2 (0-1)**	**ref 2 (1-2)**	**ref 3 (0-1)**
**mat 1 (0-1)**														
**mat 1 (1-2)**	62													
**mat 2 (0-1)**	70	64												
**mat 2 (1-2)**	64	66	65											
**mat 3 (0-1)**	69	55	73	56										
**mat 3 (1-2)**	62	60	70	62	64									
**mat 4a (0-1)**	68	61	71	60	63	64								
**mat 4b (0-1)**	63	57	69	60	62	68	76							
**mat 4c (0-1)**	62	58	59	57	59	60	71	67						
**ref 1 (0-1)**	49	64	51	59	44	50	48	47	47					
**ref 1 (1-2)**	36	43	38	48	37	41	36	38	40	56				
**ref 2 (0-1)**	38	43	42	52	37	41	38	40	40	53	56			
**ref 2 (1-2)**	39	41	41	44	40	39	39	41	40	44	46	47		
**ref 3 (0-1)**	54	50	52	49	54	47	50	49	52	41	36	39	37	
**ref 3 (1-2)**	48	53	52	57	47	48	46	47	47	56	55	59	53	47
