# Supplementary material for: Distribution and Composition of Thiotrophic Mats in the Hypoxic Zone of the Black Sea (150–170 m Water Depth, Crimea Margin)
Source: Front Microbiol. 2016 Jun 29;7:1011. doi: 10.3389/fmicb.2016.01011 (PMC4925705; doi:10.3389/fmicb.2016.01011)
Supplement: Supplementary file 2 [file Table_2.PDF]

Supplementary Table 2. Number of sequence reads and $OTU_{0.03}$ abundance for bacterial and archaeal MPTS data (without singletons)

**Sampling site ID**	**Number of bacterial sequences**	**Number of bacterial $OTU_{0.03}$**	**Number of archaeal sequences**	**Number of archaeal $OTU_{0.03}$**
**mat 1 (0-1)**	5240	1008	7024	91
**mat 1 (1-2)**	4194	985	5424	252
**mat 2 (0-1)**	12300	1320	4156	233
**mat 2 (1-2)**	5120	1127	5859	173
**mat 4 (0-1)**	6733	857	-	-
**ref 1 (0-1)**	4081	906	3524	161
**ref 1 (1-2)**	8477	1214	7518	366
**ref 2 (0-1)**	7378	914	3017	104
**ref 2 (1-2)**	1096	1025	2569	94
