# Supplementary material for: Distribution and Composition of Thiotrophic Mats in the Hypoxic Zone of the Black Sea (150–170 m Water Depth, Crimea Margin)
Source: Front Microbiol. 2016 Jun 29;7:1011. doi: 10.3389/fmicb.2016.01011 (PMC4925705; doi:10.3389/fmicb.2016.01011)
Supplement: Supplementary file 3 [file Image_1.PDF]

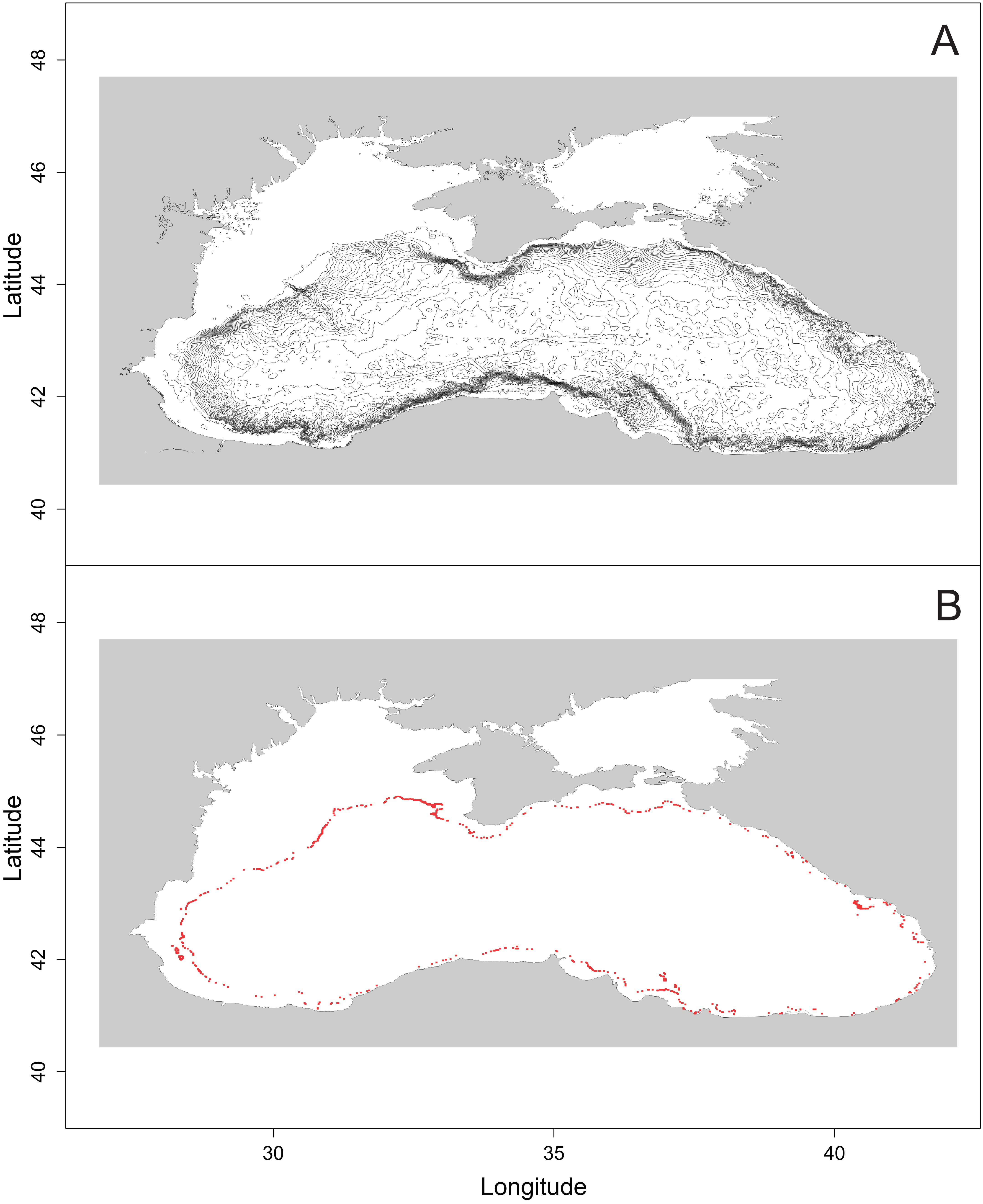

Supplementary Figure 1. Suggested distribution of thiotrophic mats around the Black Sea margin, (A) Bathymetric chart showing isobaths (contour interval 10m); (B) Area (red) based on the position of the chemocline between 150-170 m depth, calculated and visualized with the R package "MarMap" (Pante and Simon-Bouhet, 2013).
