# Supplementary material for: Distribution and Composition of Thiotrophic Mats in the Hypoxic Zone of the Black Sea (150–170 m Water Depth, Crimea Margin)
Source: Front Microbiol. 2016 Jun 29;7:1011. doi: 10.3389/fmicb.2016.01011 (PMC4925705; doi:10.3389/fmicb.2016.01011)
Supplement: Supplementary file 4 [file Image_2.PDF]

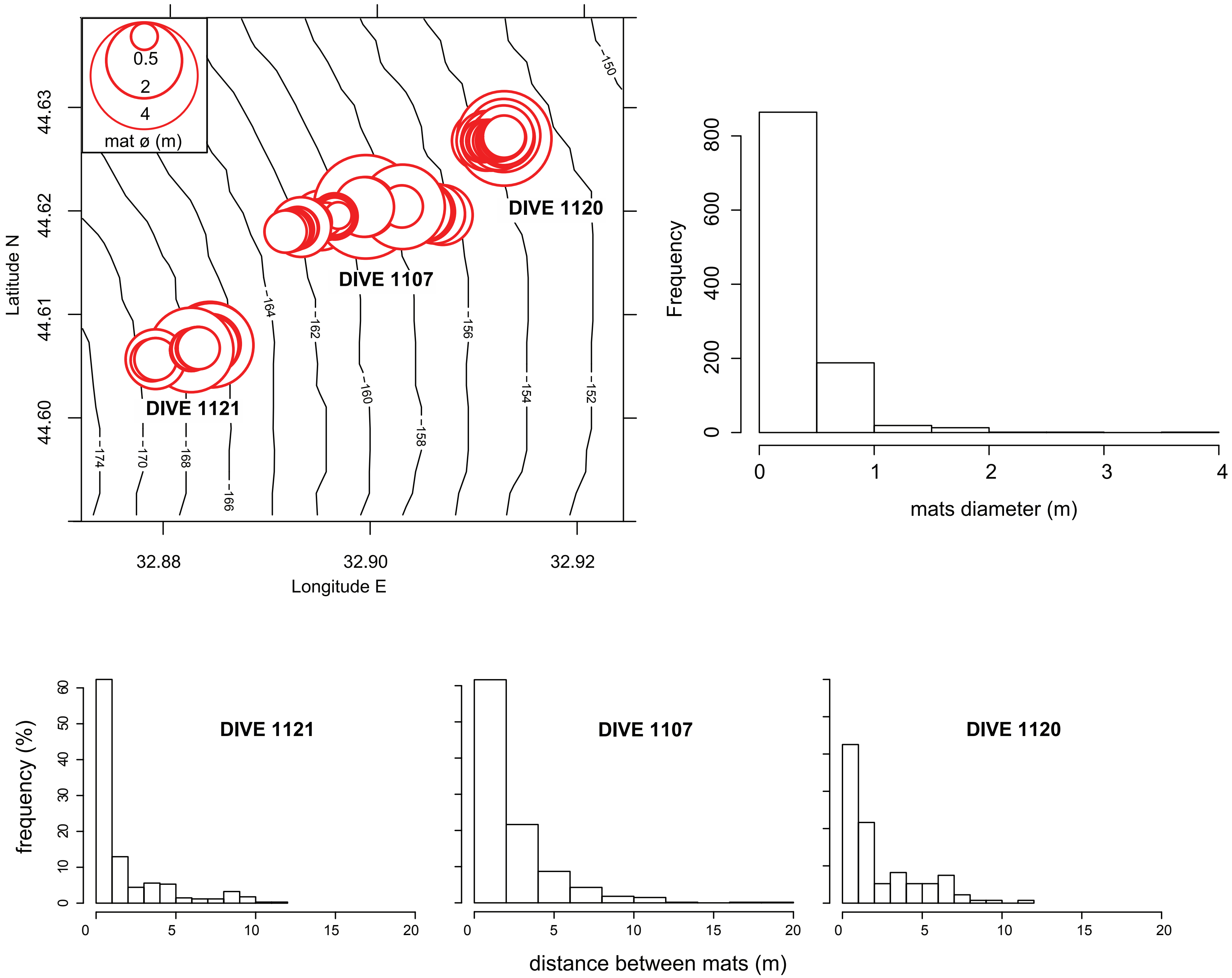

Supplementary Figure 2. Study area depicting the zone of occurrence of microbial mats (above left, the red circles were scaled to the diameter of the mats in meters) and frequency according to diameter (m) (above right); and distance between microbial mats (bottom panel) during the submersible dives (1121, 1107 and 1120).
