# Supplementary material for: Distribution and Composition of Thiotrophic Mats in the Hypoxic Zone of the Black Sea (150–170 m Water Depth, Crimea Margin)
Source: Front Microbiol. 2016 Jun 29;7:1011. doi: 10.3389/fmicb.2016.01011 (PMC4925705; doi:10.3389/fmicb.2016.01011)
Supplement: Supplementary file 5 [file Image_3.PDF]

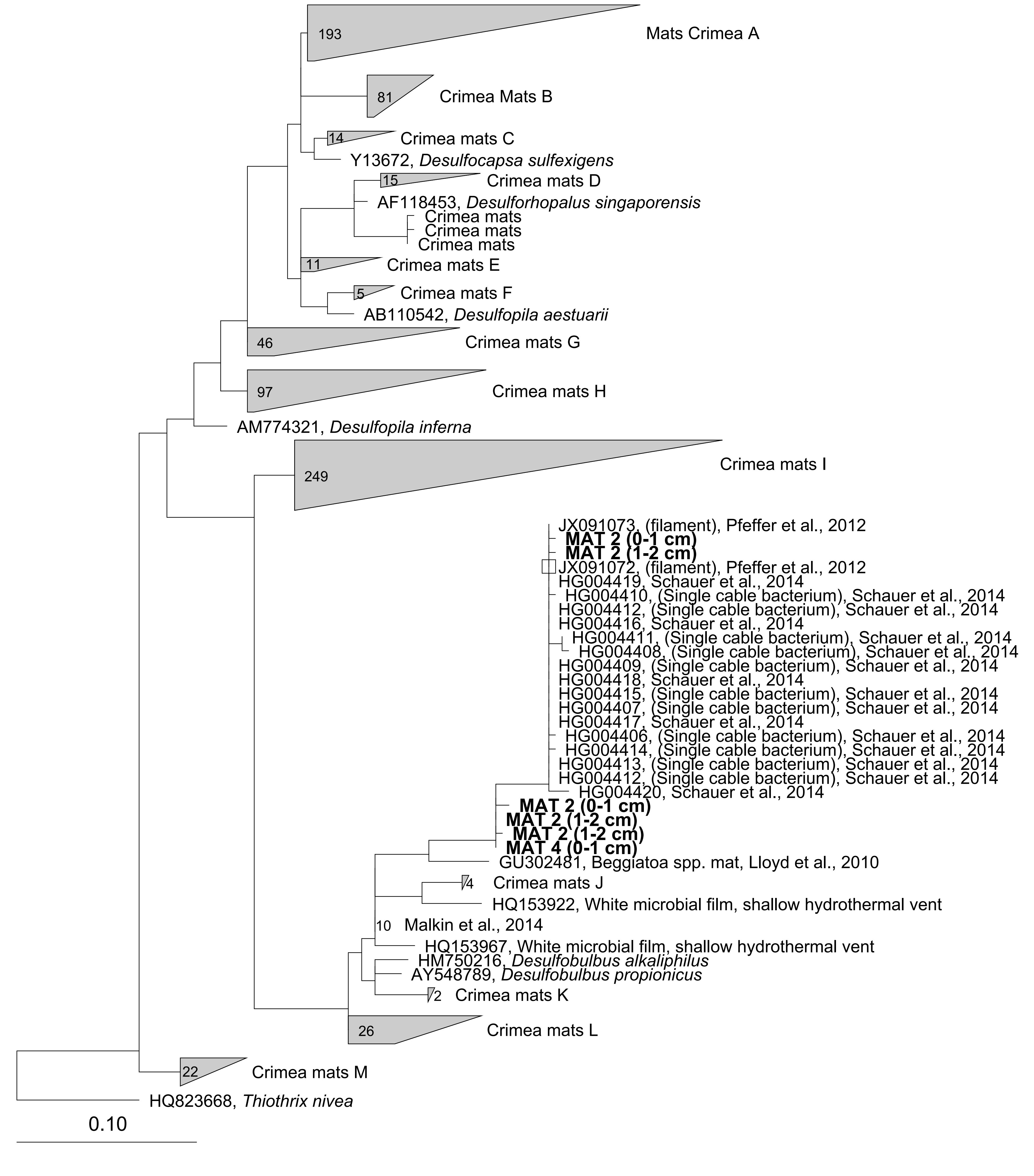

Supplementary Figure 3. Phylogenetic 16S rRNA gene based tree showing the affiliation of sequences extracted from mat-covered sediments of the Crimea margin (“Crimea mats”) with sequences recovered from sediments and assigned to the filamentous “cable” bacteria known for long-range electron transport (Pfeffer et al., 2012; Malkin et al., 2014; Schauer et al., 2014). Mats and references sequences were screened for relatives to filamentous “cable” bacteria and extracted from the data set using the “grep” function in R. Initial tree reconstruction was conducted with nearly full-length sequences of *Desulfobulbaceae* (1200 - 1400 bp). Partial sequences (this study, Pfeffer et al, 2012, Malkin et al., 2014 and Schauer et al., 2014) were subsequently inserted into the reconstructed consensus tree by applying the parsimony criteria. Only selected sequences are shown. The scale bar corresponds to 10% estimated sequence divergence. Phylogenetic classification was carried out with the software package ARB (Ludwig et al., 2004) based on the SILVA database (SSURef v119, release date: 24 July 2014). Phylogenetic tree was calculated with the maximum likelihood algorithm PHYML (100 bootstraps) as implemented in ARB using a positional variability filter.
