# Supplementary material for: Distribution and Composition of Thiotrophic Mats in the Hypoxic Zone of the Black Sea (150–170 m Water Depth, Crimea Margin)
Source: Front Microbiol. 2016 Jun 29;7:1011. doi: 10.3389/fmicb.2016.01011 (PMC4925705; doi:10.3389/fmicb.2016.01011)
Supplement: Supplementary file 6 [file Image_4.PDF]

Supplementary methods

Distribution and composition of thiotrophic mats in the hypoxic zone of the Black Sea (150-170m water depth, Crimea margin)

454 MPTS sequence processing routine

Processing of the 454 pyrosequencing amplicons was performed according to the following routine modified from Pat Schloss (Schloss et al., 2009 and 2011).

#####

Step 1 - Extract data from binary file
sffinfo(sff=Data.sff, flow=T)
Step 2 – Separate flowgrams
trim.flows(flow=Data.flow, oligos=Data.oligos, pdiffs=2, bdiffs=1, processors=16)
Step 3 – Reducing sequencing error
shhh.flows(file=Data.flow.files, processors=16)
Step 4 - Concatenate single files to make one file containing all samples:
cat *.trim.shhh.fasta > AllArch.fasta
cat *.trim.shhh.names > AllArch.names
cat *.trim.shhh.groups > AllArch.groups
Step 5 - Trim sequences using minlength=75 (for bacteria), 80 (for archaea):
trim.seqs(fasta=All.fasta,name=All.names,oligos=Data.oligo,pdiffs=1,bdiffs=0,maxhomp=8,minlength=80,flip=F, processors=16)
Step 6 - Find unique sequences:
unique.seqs(fasta=All.trim.fasta, name=All.trim.names)
Step 7 - Align sequences:
align.seqs(fasta=All.trim.unique.fasta, reference=silva.archaea.fasta, processors=16)
#for bacteria use (silva.bacteria.fasta)
Step 8 - Screen sequences:
screen.seqs(fasta=All.trim.unique.align,name=All.trim.names,group=All.groups,optimize=start-end,criteria=90)
Step 9 - Filter dataset:
filter.seqs(fasta=All.trim.unique.good.align, vertical=T, trump=., processors=16)
Step 10 - Simplify the dataset:
unique.seqs(fasta=All.trim.unique.good.filter.fasta, name=All.trim.good.names)
Step 11 - Precluster:
pre.cluster(fasta=All.trim.unique.good.filter.unique.fasta,name=All.trim.unique.good.filter.names,group=All.good.groups,diffs=0)
Step 12 - Chimera check:

chimera.uchime(fasta=All.trim.unique.good.filter.unique.precluster.fasta,name=All.trim.unique.good.filter.unique.precluster.names,group=All.good.groups, processors=16)
Step 13 - Chimera removal:
remove.seqs(accnos=All.trim.unique.good.filter.unique.precluster.uchime.accnos,fasta=All.trim.unique.good.filter.unique.precluster.fasta,name=All.trim.unique.good.filter.unique.precluster.names,group=All.good.groups)
Step 14 - Classify sequences:
classify.seqs(fasta=All.trim.unique.good.filter.unique.precluster.pick.fasta,template=nogap.archaea.fasta,taxonomy=silva.archaea.silva.tax) # for bacteria use nogap.bacteria.fasta,taxonomy=silva.bacteria.silva.tax
Step 15 - Remove lineages:
remove.lineage(fasta=All.trim.unique.good.filter.unique.precluster.pick.fasta,name=All.trim..unique.good.filter.unique.precluster.pick.names,group=All.good.pick.groups,taxonomy=All.trim.unique.good.filter.unique.precluster.pick.silva.taxonomy,taxon=Bacteria) # or remove Eukarya/Archaea
Step 16 - Simplify names:
good.filter.unique.precluster.pick.silva
system(cp All.trim.unique.
All.taxonomy)
system(cp All.trim.unique.good.filter.unique.precluster.pick.pick.fasta All.fasta)
system(cp All.trim.unique.good.filter.unique.precluster.pick.pick.names All.names)
system(cp All.good.pick.pick.groups All.groups)
Step 17 - Distance matrix:
dist.seqs(fasta=All.fasta, cutoff=0.15, proccessors=16)
Step 18 - Cluster:
cluster(column=All.dist,name=All.names)
Step 19 - Get data for 0.03:
make.shared(list=All.an.list, group=All.groups, label=0.03)
Step 20 - Consensus taxonomy for each OTU:
classify.otu(list=Alll.an.list, name=All.names, taxonomy=All.taxonomy, label=0.03, cutoff=80)

#########

Schloss, P.D., Gevers, D., and Westcott, S.L. (2011) Reducing the effects of PCR amplification and sequencing artifacts on 16S rRNA-based studies. PLoS One 6: e27310.

Schloss, P.D., Westcott, S.L., Ryabin, T., Hall, J.R., Hartmann, M., Hollister, E.B., et al. (2009) Introducing mothur: open-source, platform-independent, community-supported software for describing and comparing microbial communities. Appl. Environ. Microbiol. 75: 7537–41.
